# Supplementary material for: Advances in global sensitivity analyses of demographic-based species distribution models to address uncertainties in dynamic landscapes
Source: PeerJ. 2016 Jul 19;4:e2204. doi: 10.7717/peerj.2204 (PMC4958004; doi:10.7717/peerj.2204)

**Appendix B: Details of GRIP 2.0 Landscape Generator and Sensitivity Analysis Protocol**

*Number of populations.* The number of populations for each replicate landscape in the sensitivity analysis is sampled from a normal distribution with the mean equal to the original number of populations and a 50% coefficient of variation (CV). This default value for CV ensures a wide range of variation in number of populations to evaluate influence, and identify potential threshold effects on model predictions. As with all distributions specified in the original version of GRIP 2.0, this may be modified to reflect a species’ biology or context-specific information. If the new number of populations is less than the original model, a random subset of populations is removed from the original raster map. If the new number of populations is greater, the original populations are retained and new populations are randomly added to the original raster map, where the centre (raster) cell of a new patch does not fall on a cell currently identified as habitat. GRIP 2.0 allows users to specify a supplementary landscape mask map, used to identify regions where new patches cannot be located. This mask map may represent for example, aquatic regions for a terrestrial species or a road network. The size (i.e. area) of the new patch is varied according to a normal distribution based on the mean and standard deviation of patch sizes within the reference landscape. For the whitebark pine model, we assumed that new patches added to the landscape were circular in shape to take advantage of existing functionality of R-spatial packages, but users could customize the code to create patches with different sizes and shapes.

*Patch size.* GRIP 2.0 randomly increases or decreases the size of all patches in the reference landscape. Each patch within a replicate landscape changes in size in the same direction (i.e. either an increase or decrease in size). The proportional change in patch size is sampled from a uniform distribution with a minimum of -0.5 and a maximum value of 0.5. For each patch, an edge detection algorithm randomly selects a cell on the perimeter of a patch. Depending on the number of cells added to or subtracted from a cell, a rectangular shape is created around the selected edge cell. If a patch increases, the portion of the rectangle located outside the patch boundaries will become part of the patch structure. Otherwise, the portion of the rectangle overlapping with the patch will be removed, thereby decreasing a patch in size (Figure 1 in Appendix B).

*Habitat suitability values.* Habitat suitability (HS) values were simulated by drawing a value for each grid cell from a normal distribution based on the mean and standard deviation of HS values within the reference landscape. The default setting in GRIP 2.0 includes spatial auto-correlation among the sampled HS values. The degree of autocorrelation among HS values is based on an autocorrelated surface simulated using the RandomFields package for R {Schlather, 2013 #1092}. A Gaussian model of spatial autocorrelation was applied using a mean of 0, variance of 5, nugget value of 1, and a scale of 10, creating a generally highly correlated surface. All HS values are set to be equal to or greater than the newly sampled HS threshold value (see below). If the reference landscape contains cells outside of patches (i.e. with values < HS threshold), the HS values of these cells remain unchanged in the replicate landscape map.

*Habitat suitability threshold.* Suitable areas are those that support survival and/or reproduction (Akçakaya and Root 2005). The HS threshold value is the minimum suitability value that distinguishes between unsuitable areas (HS value < HS threshold) and suitable areas (HS value ≥ HS threshold). The threshold value is sampled applying the original threshold as a mean and a 10% CV.

*Neighborhood distance*. This is the maximum distance between patches of suitable habitat across which a population is still assumed to be panmictic (Akçakaya and Root 2005). It is used in conjunction with the habitat suitability threshold in RAMAS Spatial to identify spatially discrete patches of suitable habitat. The new distance is sampled from a normal distribution with the mean equal to the original neighborhood distance and a CV of 10%.

Demographic parameters

If initial abundances and carrying capacities for each population result from a function specified in RAMAS Spatial (as described above for whitebark pine), the values for each population are randomly sampled using the initial abundance or carrying capacity value specified for that population as the mean with a 10% CV applied. Otherwise, if a constant was specified, the new value will be sampled using the mean and standard deviation of initial abundances across all populations; a 10% CV is applied if initial abundances across all populations are identical. The remaining parameters, which include stage-specific survival and fecundity rates, among-population correlation in demographic rates, among-population connectivity, dispersal rates, dispersal survival, catastrophe probability of occurrence, catastrophe spatial extent, catastrophe multiplier (intensity), and maximum population growth rate (R_max_), and are varied in a similar manner to GRIP 1.0, a version of this freeware developed for spatial PVAs that are not habitat-based ([Curtis and Naujokaitis-Lewis, 2008](#_ENREF_14)). We provide a brief overview of the parameters varied including sampling distributions below, however, for a detailed description readers are referred to Curtis and Naujokaitis-Lewis (2008) and Appendix A and Table 1.

*Vital rates*. All non-negative vital rates are sampled using the lognormal distribution with mean = original value and SD = original value. This version of GRIP 2.0 does not vary the standard deviation of vital rates.

*Among-population correlation of vital rates.* Varies magnitude of correlations in vital rates among population pairs, sampled from a normal distribution with a mean = 0 and CV = 0.1.

*Connectivity.* To simulate dispersal barriers in the landscape, GRIP 2.0 randomly varies the total number of population pairs that will be connected through dispersal and then randomly selects which population pairs will be connected. The proportion of population pairs connected by dispersal was varied by randomly sampling the probability that any pair of populations would be connected based on a uniform distribution, where the minimum value was 0 and the maximum value was sampled from the maximum number of connections between pairs of populations.

*Dispersal rates.* Once the landscape generator portion of the sensitivity analysis is complete, dispersal rates between population pairs are recalculated based on the user-specified dispersal distance function parameters and the new distances between pairs of populations. Dispersal rates were then varied in the sensitivity analysis to where each pairwise rate between populations is modified following:

$$m_{ij}^{'}= m_{ij}+m_{ij}n$$

Where $m_{ij}^{'}$ is the adjusted dispersal rate, $m_{ij}$ is the dispersal rate between the *i*th and *j*th populations, and *n* is the modifier randomly drawn from a normal distribution (mean = 0, CV = 0.1).

*Dispersal survival*. The proportion of dispersing individuals that survive emigration to a target population (dispersal survival) is sampled from a uniform distribution varying from 0 to 1. Dispersal survival is assumed to be identical for all populations, and is modeled by creating a sink population where all emigrating individuals are contained.

*Catastrophes.* Catastrophe parameters are only varied if the original model includes a catastrophe. Parameters varied related to catastrophes include catastrophe extent, catastrophe probability, and catastrophe intensity. Catastrophe extent is a discrete variable representing whether catastrophes are local or regional in extent. The probability of catastrophe occurring is randomly varied using a normal distribution where the mean = original value with a 10% CV. Catastrophe intensity The intensity of catastrophes is varied by manipulating stage-specific or population-specific multipliers that can affect carrying capacities, abundances, vital rates, and dispersal rates. This magnitude of catastrophe effect was randomly varied using a normal distribution with mean representing the multiplier for each affected stage-specific rate and a 10% CV, in the case of the whitebark pine model where catastrophes were stage-specific.

**Appendix B Figure 1. Conceptual representation of the habitat patch change algorithm.**

Figure. Conceptual representation of the habitat patch change algorithm. A) A hypothetical landscape consisting of 2 patches, B) if patches increase in size, X indicates the cell randomly selected on which the patches ‘grow’, the mottled regions indicate the new cells added to the original patches, and C) if patches decrease in size, X marks the cell randomly selected on which the patches are decreased, with the white regions representing the cells removed from the original patch.


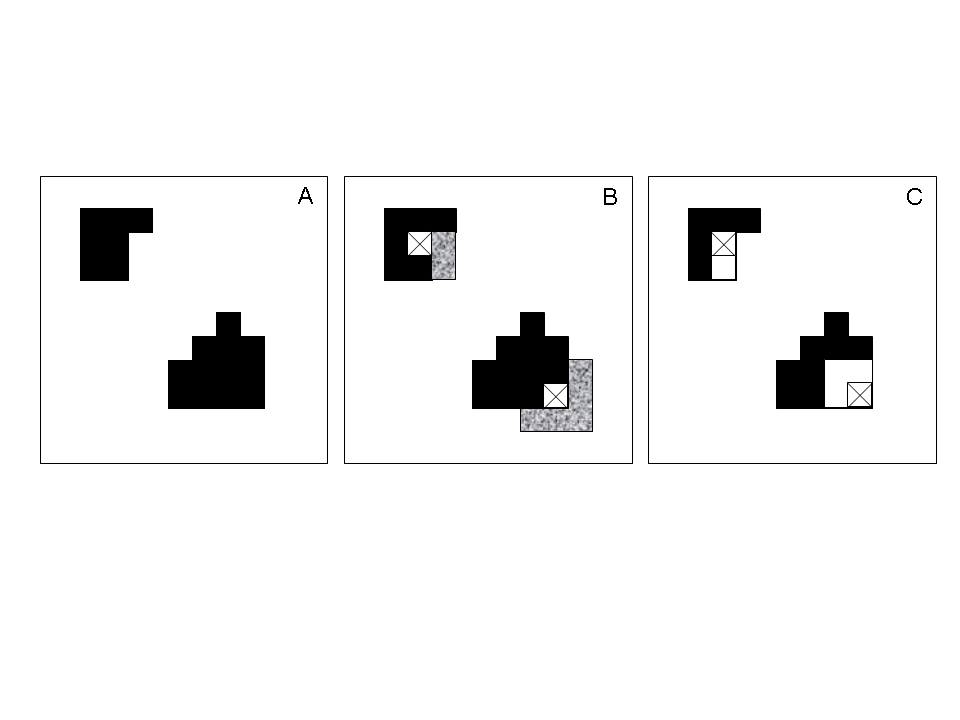

Supplement: Appendix S2 [file peerj-04-2204-s002.docx]
